# Host seeking parasitic nematodes use specific odors to assess host resources

**DOI:** 10.1038/s41598-017-06620-2

**Published:** 2017-07-24

**Authors:** Tiffany Baiocchi, Grant Lee, Dong-Hwan Choe, Adler R. Dillman

**Affiliations:** 1 Department of Nematology, University of California, Riverside, California 92521 USA; 2 Department of Entomology, University of California, Riverside, California 92521 USA

## Abstract

Entomopathogenic nematodes (EPNs) are insect parasites used as biological control agents. Free-living infective juveniles (IJs) of EPNs employ host-seeking behaviors to locate suitable hosts for infection. We found that EPNs can differentiate between naïve and infected hosts, and that host attractiveness changes over time in a species-specific manner. We used solid-phase microextraction and gas chromatography/mass spectrometry to identify volatile chemical cues that may relay information about a potential host’s infection status and resource availability. Among the chemicals identified from the headspace of infected hosts, 3-Methyl-2-buten-1-ol (prenol) and 3-Hydroxy-2-butanone (AMC) were selected for further behavioral assays due to their temporal correlation with the behavioral changes of IJs towards the infected hosts. Both compounds were repulsive to IJs of *Steinernema glaseri* and *S*. *riobrave* in a dose-dependent manner when applied on an agar substrate. Furthermore, the repulsive effects of prenol were maintained when co-presented with the uninfected host odors, overriding attraction to uninfected hosts. Prenol was attractive to dauers of some free-living nematodes and insect larvae. These data suggest that host-associated chemical cues may have several implications in EPN biology, not only as signals for avoidance and dispersal of conspecifics, but also as attractants for new potential hosts.

## Introduction

**Figure 1:**
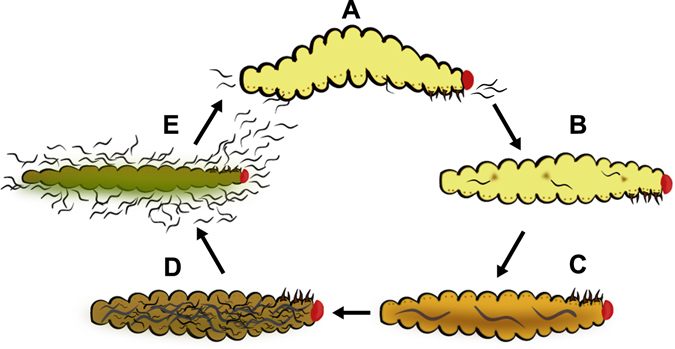
The EPN life cycle. (**A**) Uninfected host is infected with infective juveniles (IJs). (**B**) IJs invade and release symbiotic bacteria. (**C**) The bacteria proliferates and IJs mature into adulthood. (**D**) Adults produce progeny. (**E**) Eventually resources run out and newly emerging IJs disperse from depleted cadaver.

Entomopathogenic nematodes (EPNs) are insect-killing parasites used in biological control and are a model system for studying host-parasite interactions. EPNs can infect and kill a host within 48 hours and are commercially available for use in home gardens and industrial agriculture^1, 2^. In the EPN life cycle, free-living infective-juveniles (IJs) encounter a host (either uninfected or possibly at an early stage of infection) (Fig. 1A), and then decide whether or not to invade the host. Once an IJ invades a host it releases symbiotic bacteria (Fig. 1B), which proliferate and help to both kill the host and provide a food source for the growing nematodes^3^ (Fig. 1C).

*Steinernema* spp. are gonochoristic, requiring a male and female in order to produce offspring^4^. Within the host approximately 2–3 generations of nematodes can be produced (Fig. 1D), but eventually resources begin to run out inside the insect cadaver^2^. As resources are depleted, the L2 juveniles will associate once more with the bacteria and take an alternative developmental pathway, becoming infective juveniles (IJs) rather than L3 juveniles. These IJs will then emerge and search for a new host to infect^2, 5^ (Fig. 1E). In their search for a new host there are two host-seeking strategies–employed by IJs–which represent endpoints of a continuous spectrum. The first is a cruise foraging strategy where the IJ spends the majority of its time actively moving in search of a host^6, 7^. The second strategy is an ambush foraging strategy where the IJ employs a sit-and-wait approach, waiting for a potential host to pass close by, allowing the IJ to attach and invade^7, 8^. Between these two endpoint foraging strategies there are species of EPNs that have been classified as intermediates^6, 9^. These foraging strategies are employed by individuals within a population and it has been shown that even for a species like *S*. *carpocapsae*. which is described as an ambush forager, a small proportion of individuals are cruise foragers and have been referred to as “sprinters”^8^. Here, we worked with four species of *Steinernema*: *S*. *carpocapsae*, an ambush forager^6, 8^; *S*. *glaseri*, a cruise forager^6, 9^; and *S*. *riobrave* and *S*. *feltiae*, which are described as intermediate foragers^6, 9^.

EPN foraging strategies and the decision of whether or not to infect certain hosts is informed by olfactory and mechanosensory cues. Previous work has shown that volatile odorants emitted by uninfected insects elicit host-seeking behavior in EPN IJs^10–12^. IJs rely on chemical cues in the environment to locate, identify, and evaluate their hosts^7^. It has been shown that EPNs can distinguish between uninfected and infected hosts, and even between hosts with conspecific or heterospecific infections^13^. However, the dynamics of host odorant profiles throughout the course of infection and which odorants IJs use to differentiate conspecific and heterospecific infections remains unknown. We evaluated the behavioral response of EPNs to infected hosts over time and identified odorants used by IJs to inform behavioral decisions regarding attraction to or avoidance of previously-infected hosts.

## Results

### Infective Juveniles can Differentiate Between Uninfected and Infected Hosts

We evaluated the responses of the IJs of four EPN species (*S*. *carpocapsae*, *S*. *feltiae*. *S*. *glaseri*, *and S*. *riobrave*) in response to last instar *Galleria mellonella* hosts at various stages of infection. We determined the IJ response to uninfected hosts, and hosts that had been infected for different amounts of time (1, 3, 5, 7, 9, and 16 days). To perform these evaluations, we used a 2-choice assay, designed to test the effects of volatile compounds emitted by the hosts^10, 11^.

**Figure 2:**
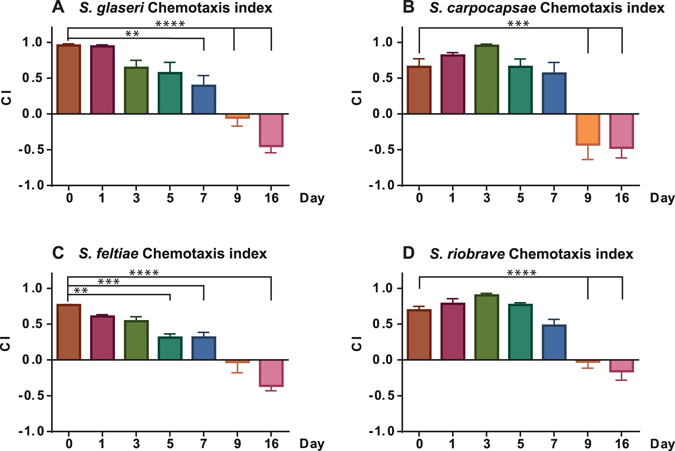
Chemotaxis indices shown for the four species of *Steinernema* EPN IJs. CI values near: +1.0 indicate high attraction, near zero indicate indifference, and near −1.0 indicate high repulsion. Statistical significance was evaluated using an unpaired, ordinary, one-way ANOVA with Tukey’s multiple comparisons post-test. Error bars represent SEM. **P < 0.01; ***P < 0.001; ****P < 0.0001.

Our results showed that all four species of EPNs tested were attracted to the odors of uninfected *G*. *mellonella* (Fig. 2A–D). By 9 days post-infection (dpi), attraction had been abolished, and the IJs of all four species were repelled from cadavers by 16 dpi. At late stages of infection such as 9 and 16 dpi, the resources are often depleted and the cadaver contains IJs ready to emerge in search of new hosts^14^.

### IJs Respond to Infected Cadavers in a Species-Specific Manner

IJs of *S*. *glaseri*, *S*. *carpocapsae*, *S*. *feltiae*. *and S*. *riobrave* can differentiate between uninfected and infected hosts, however, the trend of host-seeking behavior varied in a species-specific manner (Fig. 2A–D). There were two clear trends: Steady decrease in attraction, or an initial increase, followed by a decrease in attraction. The host-seeking behavior observed for *S*. *glaseri* and *S*. *feltiae* revealed that these species were most attracted to uninfected hosts, and exhibited a general trend of reduced attraction by 3 dpi. The reduction in attraction continued over the course of the infection and by 9 dpi attraction had been fully abolished, while by 16 dpi repulsion was observed (Fig. 2A,C).

*Steinernema carpocapsae* and *S*. *riobrave* exhibited attraction to uninfected hosts, however the highest attraction observed was towards recently deceased cadavers at 3 dpi. The attraction then declined over the later stages of infections until repulsion was observed at 16 dpi.

### Some EPNs Chemotax More Readily Than Others

**Figure 3:**
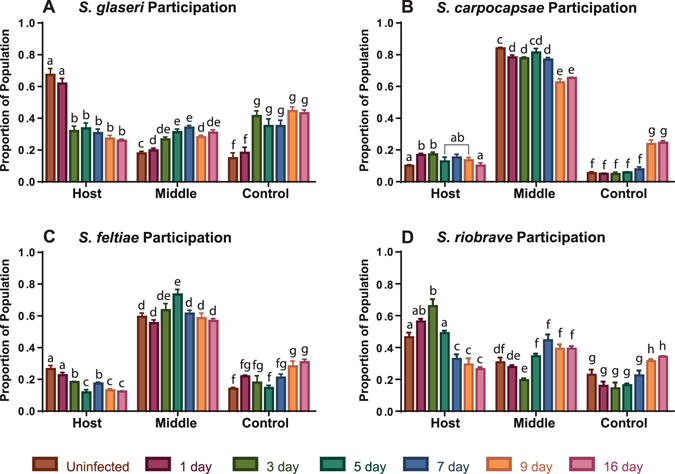
Participation values for four species of *Steinernema* EPN IJs. Participation values were derived from separating the plate into three sections and scoring nematodes that had moved directionally 1 cm either towards the side where host volatiles or control air was being delivered. Those that did not move directionally out of the center (by at least 1 cm) were scored as remaining in the middle. Statistical analysis was done using an unpaired, ordinary one-way ANOVA evaluation for data points within–but not between–each group of “Host”, “Middle”, and “Control”. Bars with the same letter values are not significantly different. For breakdown of the scoring template please see Fig. 4. Error bars represent SEM.

**Figure 4:**
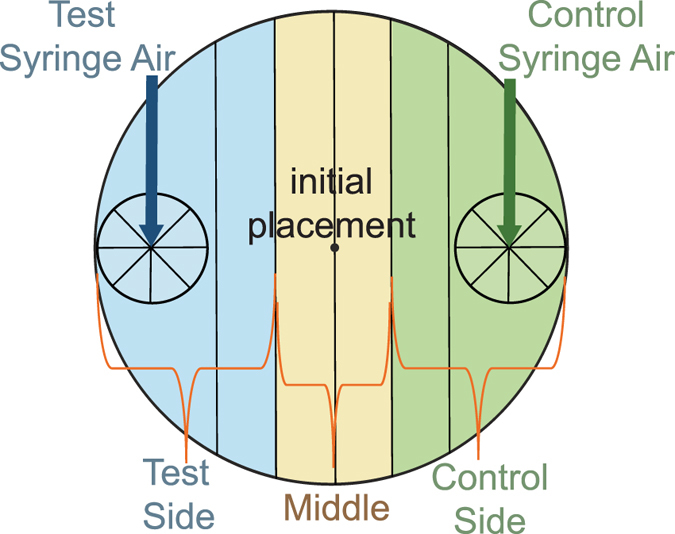
Template used in chemotaxis assays. The scoring circles indicate where volatiles were delivered (for insect-odor response assays) or where odorants were applied (for chemical response assays). Scoring circles were used to count nematodes and calculate chemotaxis indices (CI values), while the 3 designated sides (test, middle and control) were used to determine participation.

Although chemotaxis indices are robust and informative^10, 11, 15^, we observed interesting patterns of IJ participation that were not captured using a chemotaxis index (CI) (Fig. 3A–D). To better characterize the movements of the population of IJs in the testing arena, we divided our scoring templates into three sections (Fig. 4). Nematodes that moved out of the initial placement (center region) were scored as moving either toward the host or toward the control. The nematodes that remained close to the center of the arena were counted as remaining in the middle section (i.e. had not moved directionally).

For *S*. *glaseri* we observed the highest participation of IJs in chemotactic behavior in response to uninfected hosts, where 82.1% of the IJs were moving away from their initial placement (Fig. 3A). This is calculated by combining the proportion of the population moving towards the host side (67.3%) and the proportion moving towards the control side of the plate (14.8%). The lowest participation of *S*. *glaseri* IJs was in response to 7 dpi cadavers, where we observed 69.5% of the population moving directionally. On average we observed 73% of *S*. *glaseri* IJs moving directionally across all time points. This indicates that the chemotaxis data is representative of the majority of the population and that most *S*. *glaseri* IJs participate in chemotaxis behavior.

In contrast to *S*. *glaseri*, *S*. *carpocapsae* IJs demonstrated poor participation in chemotaxis assays (Fig. 3B). Participation was highest in response to 9 dpi cadavers, yielding a participation of 37.4%. The lowest participation value exhibited by *S*. *carpocapsae* IJs was in response to uninfected hosts, where only 15.6% participated in chemotaxis. On average we observed an overall participation of only 24.8% across all time points tested. These data support the description *of S*. *carpocapsae* as an ambush forager and supports previous claims that *S*. *carpocapsae* does not respond well to uninfected hosts, as measured by chemotaxis^13, 16^. Although we did record robust CI data for *S*. *carpocapsae*, the participation data reveal that the CI measures only a small proportion of the population, likely the “sprinters” previously described^8^.

We found that *S*. *feltiae*. despite being classified as an intermediate forager, behaves more like an ambush forager (Fig. 3C). Although the general trends in attraction differ between *S*. *feltiae* and *S*. *carpocapsae*, we found that on average the participation of *S*. *feltiae* is lower than that of either *S*. *riobrave* or *S*. *glaseri*. We found that *S*. *feltiae* exhibited highest attraction to hosts at 1dpi, with 44.6% of the population moving directionally. The lowest participation we observed for *S*. *feltiae* was in response to 5 dpi cadavers, where only 26.5% of the population moved directionally. The average participation for *S*. *feltiae* was only 38.7% across all time points. This indicates that while more *S*. *feltiae* IJs participate in chemotaxis than *S*. *carpocapsae* IJs, the CIs represent slightly more than one third of the population. These data suggest that within the spectrum of cruise and ambush foraging strategies, *S*. *feltiae* behaves more like an ambusher.

We found that *S*. *riobrave* IJs had high participation in chemotaxis assays, only slightly lower than *S*. *glaseri* (Fig. 3D). For *S*. *riobrave* maximum participation was 80.5% of the population in response to cadavers at 3 dpi. The lowest observed participation was 55.4% in response to cadavers at 7 dpi, while the average participation across all time points was 66.4%. These data demonstrate that *S*. *riobrave* had participation resembling that of *S*. *glaseri*. Thus despite being classified as an intermediate forager, it could be argued that *S*. *riobrave* displays cruiser-type foraging in response to volatile host cues.

Because previous research has demonstrated that the response of nematode dauers and infective juveniles is age dependent^17, 18^, we evaluated the effects of IJ age on the responses of *S*. *glaseri* and *S*. *riobrave* to uninfected hosts and 16 dpi cadavers (Supplementary Figs S1 and S2). *Steinernema glaseri* and *S*. *riobrave* were selected for evaluation due to their high level of participation. We found that the response of *S*. *glaseri* IJs to uninfected hosts was not age dependent (Fig. S1). In response to 16 dpi cadavers we observed that older IJs were not as repulsed by the odors, though this observation was not statistically significant (Fig. S2). The participation of *S*. *glaseri* IJs in chemotactic behaviors in response to 16 dpi cadavers did not differ significantly between age groups. However, general trends revealed slight increases in the number of IJs heading towards 16dpi cadaver odors and those IJs remaining in the middle, along with a slight decrease in the proportion of IJs traveling towards the control side of plate (Fig. S1).

*Steinernema riobrave* IJs did not exhibit any significant age-dependent shifts in chemotaxis index values in response to either uninfected or 16 dpi cadavers (Supplementary Fig. S3). We did observe an increase in older IJs traveling towards the control side of the plate in response to uninfected hosts, but there were no other significant shifts in proportion of IJs heading towards the host odors or remaining in the middle in response to uninfected host odors (Fig. S3).

### A Diverse Array of Odorants Influence Host-Seeking Behavior

Having identified discrete changes in host-seeking behavior throughout the course of infection, we next worked to identify odorants that might mediate these changes in behavior. To address this we evaluated the headspace of *G*. *mellonella* hosts, both uninfected and infected with either *S*. *glaseri* or *S*. *riobrave*, using solid phase microextraction (SPME) followed by gas chromatography and mass spectrometry (GC-MS).

Our aim was to identify odorants that might be responsible for the behavioral shifts we observed in EPN IJs. We sampled the headspace of uninfected and infected insects at 1, 3, and 16 dpi. The uninfected and 1 dpi time points were associated with attraction for both *S*. *glaseri and S*. *riobrave* (Fig. 2A,D). Whereas recently deceased hosts, at 3 dpi, were associated with a general increase in attraction for *S*. *riobrave* and a general decrease in attraction for *S*. *glaseri* (Fig. 2A,D). Lastly, 16 dpi was associated with IJ repulsion. We expected that odorants present in the headspace of uninfected hosts might decrease in abundance or disappear, while other chemicals appeared and increased in abundance during the progression of the infection.

From our headspace analysis we found several chemicals: α-pinene, β-pinene, nonanoic acid, butanoic acid, and longifolene, which had been previously associated with uninfected *G*. *mellonella* hosts^10, 11, 19^. However, one of our controls–SPME-GC-MS experiments examining the odors emitted by the pine chips used as packing material for *G*. *mellonella*–showed that the likely source of these odorants was the wood chips rather than the larvae.

**Figure 5:**
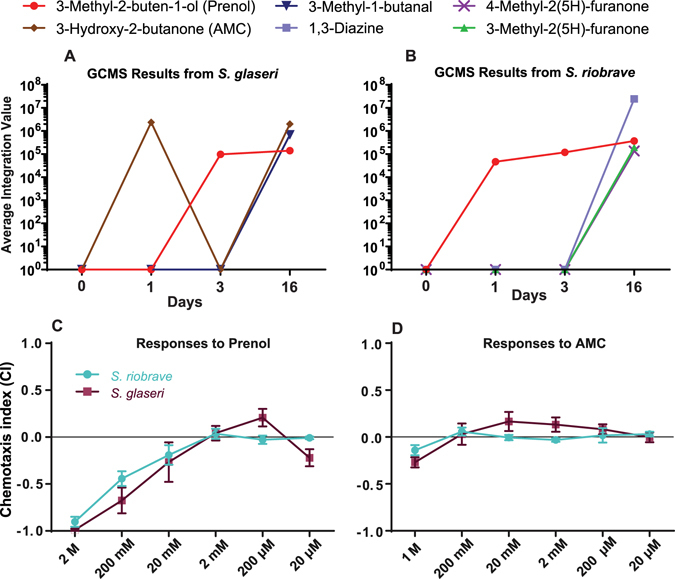
Panels A,B: Results from GC-MS of (**A**) *S*. *glaseri* and (**B**) *S*. *riobrave* infected *G*. *mellonella* hosts. Numbers represent integration values of peaks. This reflects the overall relative abundance of individual odors. Here we found that most odors are appeared in highest abundance at 16 days post infection (dpi), however two chemicals–AMC and prenol–stood out by appearing at earlier time points. 3-Hydroxy-2-butanone (AMC) is found in association with *S*. *glaseri*-infected hosts at 1 dpi and 16 dpi. 3-Methyl-2-buten-1-ol (prenol) is found in both *S*. *riobrave* and *S*. *glaseri* infected hosts, and appears at 1dpi (for *S*. *riobrave*-infected hosts only) as well as at 3 dpi, and 16 dpi in both *S*. *riobrave* and *S*. *glaseri*-infected hosts. Panels C,D: Results of dose response curves for (**C**) 3-Methyl-2-buten-1-ol (prenol) and (**D**) 3-Hydroxy-2-butanone (AMC). These show the responses of both *S*. *glaseri* and *S*. *riobrave* IJs to various concentrations of these chemicals. It is worth noting that the concentrations listed are what was applied to the experimental arena, but are certainly higher than the concentration experienced by the nematodes. Error Bars represent SEM.

Using GC-MS we identified 3 consistent odorants from *S*. *glaseri*-infected hosts, and 4 consistent odorants from *S*. *riobrave-*infected hosts. From *S*. *glaseri-*infected larvae we found 3-Methyl-2-buten-1-ol, Butanal-3-methyl, and 3-Hydroxy-2-butanone (Fig. 5A). From *S*. *riobrave-*infected larvae we found 3-Methyl-2-buten-1-ol, 4-Methyl-2(5H)-Furanone, 3-Methyl-2(5H)-Furanone, and 1,3-Diazine (Fig. 5B). Odors were identified by comparing spectral analysis of the sampled headspace against chemical profiles in a database. The majority of the odorants we found came from 16 dpi cadavers, and all of the odorants identified were found at highest relative abundance at this time point (Fig. 4A,B).

Two odorants, 3-Methyl-2-buten-1-ol (prenol), and 3-Hydroxy-2-butanone (acetyl methyl carbinol or AMC), were chosen for behavioral evaluation due to their appearances at time points associated with IJ attraction (i.e. 1 dpi –3 dpi cadavers) as well as time points associated with repulsion (i.e. 16 dpi cadavers). All other odorants appeared only on 16 dpi. We reasoned that AMC and prenol were the odorants most likely to elicit dose-dependent behaviors from IJs due to their appearance at the time points associated with both attraction (1–3 dpi) and repulsion (16 dpi).

Both AMC and prenol retention times were compared to synthetic standards to confirm retention time matches (see methods information for standards used). AMC (3-Hydroxy-2-butanone) was chosen for appearance at both 3 dpi–a time point associated with IJ attraction and 16 dpi–a time point associated with IJ repulsion/ reduced attraction (Fig. 4A). However it should be noted that AMC was only present in *S*. *glaseri*-infected cadavers. Similarly, prenol (3-Methyl-2-buten-1-ol) was chosen for its appearance in both *S*. *glaseri* and *S*. *riobrave* infected cadavers. Additionally, prenol was present at time points associated with IJ attraction (1 dpi for *S*. *riobrave* and 3 dpi–for both *S*. *riobrave* and *S*. *glaseri*) as well as time points associated with IJ repulsion (16 dpi for both *S*. *riobrave* and *S*. *glaseri*.) (Fig. 4A,B).

### *S*. *glaseri* and *S*. *riobrave* IJs are Repelled by Prenol and AMC

Having identified prenol and AMC as potentially important cues for IJs, we measured the response of *S*. *glaseri* and *S*. *riobrave* IJs to these odorants. Since these chemicals were identified at time points associated with both attraction (at 1dpi and 3 dpi) and repulsion (at 16 dpi), we hypothesized that low amounts of these odorants would elicit attraction responses from EPNs, while higher amounts would elicit repulsion. Prenol elicited strong repulsion responses for both *S*. *glaseri* and *S*. *riobrave* in a dose-dependent manner (Fig. 5C), while AMC elicited mild repulsion behavior from both *S*. *glaseri* and *S*. *riobrave* in high abundance (Fig. 5D). However, we found no statistically significant difference between the responses to varying doses of AMC.

We note that although in these assays we applied doses of 2 M, 200 mM, 20 mM etc., the actual concentrations experienced by the nematodes were probably much lower than these initial concentrations, as the chemicals diffused into the surrounding air and agar. The results overall indicate that both *S*. *glaseri* and *S*. *riobrave* IJs are sensitive to prenol. It is important to note that the exact concentrations of these odors that are emitted by the hosts are not known, and can vary from host to host, as well as over time. Methods for determining ecologically-relevant concentrations have yet to be adequately established in this field, and as such we tested a range of concentrations that we thought were appropriate.

Additionally we tested whether or not IJ age would have an effect on the responses of *S*. *glaseri* and S. riobrave IJs to prenol (Supplementary Figs S1 and S3). We found that the responses by *S*. *riobrave* did not differ between younger and older IJs either as measured by chemotaxis index or participation. *S*. *glaseri* IJs did not show a significant change in chemotaxis index, but did show significant shifts in participation. We observed a significant increase in the proportion of older *S*. *glaseri* IJs that remained in the middle, and a significant decrease in the proportion of older IJs chemotaxing towards the control side of the plate (Supplementary Fig. S1). This indicates that although the overall response to prenol did not change, the sensitivity of prenol may be age dependent.

### Prenol Elicits Different Behaviors from Organisms of Different Ecological Niches

**Figure 6:**
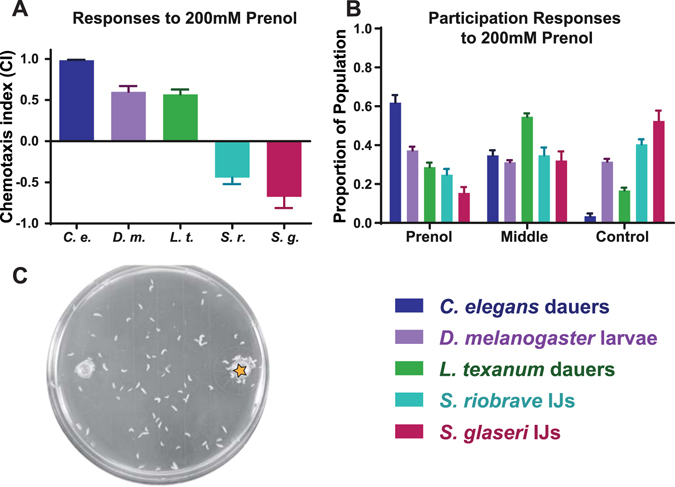
Multi-Species response to prenol. (**A**) Chemotaxis indices for *Caenorhabditis elegans* (dauers) (*C*. *e*.) *Drosophila melanogaster* (larvae) (D. m.), *Levipalatum texanum* (dauers) (L.t.), *S*. *riobrave* IJs (*S*. *r*.), *S*. *glaseri* IJs (*S*. *g*.). (**B**) The participation values of each of the species tested in (**A**). (**C**) A representative photo of *D*. *melanogaster* larvae in response to 200 mM prenol. Star indicates location of prenol (diluted to 200 mM in paraffin oil).

Of the two odorants tested, prenol elicited the strongest response (Fig. 5C). A paucity of information regarding prenol and its overall ecological relevance raises questions about how other organisms might respond to it. To gain a better understanding of the possible ecological relevance of prenol to other organisms, we evaluated the responses of dauer-stage nematodes for two free-living nematode species: *Caenorhabditis* *elegans* dauers and *Levipalatum texanum* dauers^20^. Additionally we evaluated *Drosophila melanogaster* larvae to prenol. *C*. *elegans* dauers were highly attracted to prenol and also exhibited very high participation (Fig. 6A,B). We observed that approximately 61.8% of the population of *C*. *elegans* chemotaxed towards prenol, with approximately 34.7% remaining in the middle and a mere 3.4% moving towards the control side of the plate. The attractive response of *C*. *elegans* and the association of prenol with EPN-infected cadavers merits further study. *Levipalatum* *texanum* dauers–another free-living nematode–was evaluated, and exhibited fairly high attraction to prenol (Fig. 6A). However, participation of *L*. *texanum* was much lower than that of *C*. *elegans*. Only 28.7% of the population chemotaxed towards prenol, 54.6% remained in the middle, and 16.7% moved towards the control side of the plate (Fig. 6C). *D*. *melanogaster* larvae also exhibited high attraction to prenol. However, participation data revealed that the movement of larvae overall was primarily stochastic. The pattern of attraction revealed that there was random movement by the larvae until they came within a certain range of this odorant (Fig. 6C).

### Prenol Drives EPN Repulsion From Uninfected Cadavers

**Figure 7:**
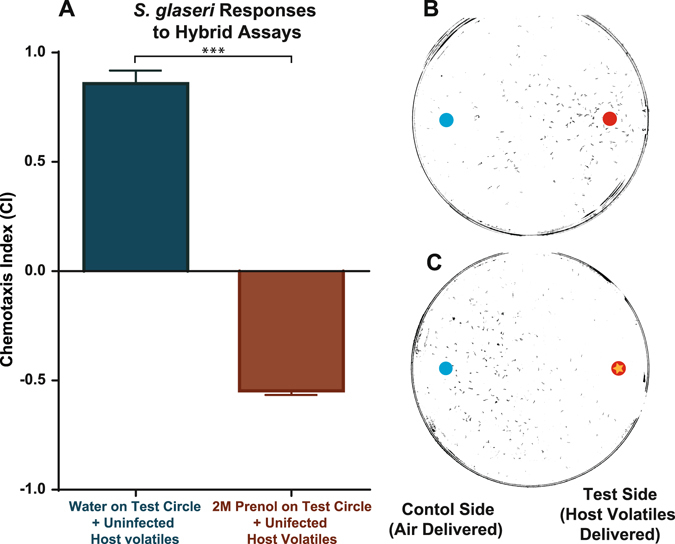
Hybrid Assay results. (**A**) Chemotaxis index for hybrid assays done with *S*. *glaseri* IJs. Statistical analysis done using a two-tailed, paired, parametric t-test. Error bars represent SEM, ***P < 0.001 (**B**) A representative photo of a control hybrid assay using volatile and soluble cues, in this experiment 5 µl of ultrapure water was placed on the agar plate underneath where uninfected host volatiles were being delivered. The blue dot (on left side of the plate) represents the location of where air from control syringe was delivered, and the red dot (on right side of the plate) represents where volatiles from uninfected hosts was delivered. (**C**) A representative photo of a hybrid assay where 5 µl 2 M prenol–diluted in ultra-pure water–was added to the test circle–where uninfected host volatiles were being delivered. The combination of prenol and uninfected host volatiles is indicated by red dot with gold star. The blue dot (on left side of plate) indicates where air from control syringe was being delivered. Error bars represent SEM. ***P < 0.001.

To evaluate the potential ecological relevance of the repulsive behavior caused by prenol, we evaluated the response of *S*. *glaseri* to uninfected host volatiles in combination with prenol. As noted above, *S*. *glaseri* is highly attracted to uninfected host volatiles (Fig. 2A). In our initial assessments we found that behavioral experiments with *S*. *glaseri* and uninfected hosts yielded a chemotaxis index of 0.955 (extremely high attraction). In contrast, when exposed to 2 M prenol, we observed near-perfect repulsion, yielding a chemotaxis index value of −0.989 (Fig. 5C). The hybrid assay set-up–using both uninfected host volatiles and prenol–revealed that prenol completely abolished *S*. *glaseri* attraction to uninfected waxworm hosts and instead caused very strong repulsion (Fig. 7A–C).

## Discussion

Our initial assessments of the data revealed that the IJs of all four EPN species were repelled by late-stage infected hosts (9 and 16 days post infection). The repulsion observed indicates that using olfaction alone, IJs can detect odors associated with late-stage-infected host cadavers–which are no longer suitable for infection. Our results suggest that the infection process leads to changes in host health such as a reduction in available resources within the host. These changes affect the suitability of a host, which can greatly influence the behavior of free-living IJs^13, 14^. Furthermore, our results support previous reports that the IJs of some EPN species are more attracted to hosts that have been recently parasitized by conspecifics, than to uninfected hosts^21^. It should be noted that previous work has shown that hosts can be infected even if the host has already been parasitized or is deceased^22^. Our results also indicate that volatile cues (including, but not limited to CO_2_) can elicit behavioral responses that change dynamically as the infection progresses^7, 10, 11^.

Our participation results supported the previous characterization that *S*. *glaseri* is a cruiser-type nematode and participates well in chemotaxis assays^14, 16, 23^. Similarly our results reinforce previous reports that *S*. *carpocapsae* IJs behave more like ambush foragers, where most of the IJs did not participate in chemotaxis behavior, at least not in the time frame of the assays we used^23^. Furthermore, we observed that while we could calculate repeatable CIs for all four species of EPNs, a large proportion of *S*. *carpocapsae* and *S*. *feltiae* IJs did not move from their starting position in our assays. This meant that the CI was based on only a small subset of the population. For *S*. *carpocapsae* in particular, the population contains “sprinters” which have been previously observed and characterized as utilizing a cruiser-type foraging strategy rather than the ambusher type strategy most *S*. *carpocapsae* IJs use^8, 24^. We believe that our CI values reflect the behaviors of these sprinters, rather than the general behaviors elicited by *S*. *carpocapsae*. Additionally, the participation measure indicated that nematodes classified as intermediates can, as a population, lean towards a particular end of the foraging spectrum, between cruise and ambush foraging. For example, *S*. *riobrave*, which is classified as an intermediate^11, 23^, exhibited behavioral responses that mirrored those of *S*. *glaseri* IJs. *S*. *feltiae* has been described as a cruise forager^25, 26^, and an intermediate forager when compared to EPNs from various genera^27^, however our results indicated that *S*. *feltiae* participation was similar to that of an ambusher type foragers such as *S*. *carpocapsae*. By evaluating participation data, we could more accurately establish where a particular species of host-seeking nematode may fall within the spectrum of cruiser to ambusher. Although the CI provides some information regarding host-seeking behavior, the resolution is limited. Adding participation data remedies this issue, and relays a clearer picture of the behavioral responses of IJs to the progression of the infection.

It is known that some behavioral responses of nematode dauers and IJs are age dependent^17, 18^. Our experiments revealed some age-dependent differences in *S*. *glaseri* IJs responding to late-stage, resource depleted cadavers, where younger IJs displayed stronger repulsion to these cadavers than older IJs. The participation data revealed a similar trend in the response of *S*. *glaseri* IJs to prenol, where a higher proportion of young IJs were repelled by prenol than older IJs. These findings lead us to speculate that younger IJs may be more sensitive to dispersal cues, which would be in high abundance in the late-stage infected cadavers, and the age-dependent participation of IJs responding to prenol support our hypothesis that prenol may be a dispersal cue.

It is worth noting that previous work has shown some behavioral trends that differ from what we have reported here. For example, Grewal *et al*.^13^ indicated that *S*. *riobrave* is more attracted to uninfected hosts than infected ones, that *S*. *glaseri* is more attracted to infected hosts than naïve hosts, and that the responses of *S*. *feltiae* towards uninfected and infected hosts did not differ. Our results suggest the contrary; we observed that *S*. *riobrave* appeared to have slightly increased attraction to hosts at 3 days post infection compared to uninfected hosts (Fig. 2B). *S*. *glaseri* was most attracted to uninfected hosts, with attraction decreasing over time (Fig. 2A). Additionally, we found that *S*. *feltiae* does exhibit altered behavior towards infected hosts, showing highest attraction to uninfected hosts, with attraction decreasing over time. These differences between our results and previously published work may be due to differences in the methods employed. Grewal *et al*. employed assays that used a static environment, they infected hosts with a higher density of nematodes, and they focused mainly on hosts infected for 4 and 24 hours^13^.

The results of our SPME-GC-MS work revealed a set of six odors, two of which we explored: Prenol (also known as 3-Methyl-2-buten-1-ol, 3,3-dimethylallyl alcohol, and 3-methyl-2-butenyl alcohol) and acetyl methyl carbinol (AMC) (also known as 3-Hydroxy-2-butanone or acetoin). The methods that we employed have been previously used with much success in identifying volatile organic compounds (VOCs) that elicit responses from various nematode species^10, 11^. However, not much work has been done to identify volatile compounds associated with the progression of an infection. This is the first report of these identified VOCs that occur in relation to nematode-infected insects. These odors were identified at infection time points associated with both IJ attraction and repulsion. Because of their appearance in early time points and gradual increase in relative abundance over time, we hypothesized that the IJs might respond to these odorants in a dose-dependent manner.

AMC has been mentioned in the literature in different contexts, some of which relate to its potential importance in EPN infections. It was identified as a volatile produced by *Zophobas morio* larvae^10^ and as a metabolic byproduct of the mammalian-parasitic nematode *Ascaris lumbrioides* under anaerobic conditions^28^. It has even been found in association with *Serratia spp*. bacteria isolated from the plant-parasitic nematode *Bursaphelenchus xylophilus* (the pine wilt nematode)^29^.

Prenol has been mentioned in association with walnut twig beetles (*Pityophthrorus juglandis*)^30^ and rectal gland secretions produced by certain male fruit flies (*Bactrocera visenda*)^31^. Prenol is an isoprenoid, formed through the mevalonate pathway used to create isoprene^32, 33^. The mevalonate pathway and the formation of isoprene are linked with insect juvenile hormone production^34, 35^ and the formation of insect pigments^36^. Additionally, isoprene may be a chemical constituent of insect epicuticle layers^37^. In addition, prenol has been found in association with cyanobacteria biofilms, and in this context it was shown that *C*. *elegans* adults were attracted to prenol (approximate CI value = 0.41)^38^. Although prenol is mentioned in a wide array of circumstances, it was not previously known to be associated with nematode-infected insects. Interestingly, a recent study has found a very similar organic compound by the name of methyl 3-methyl-2-butenoate (MMB) which closely resembles prenol in structure. MMB was found in association with a nematophagus fungus, which produces this odorant to attract adult hermaphrodite *C*. *elegans*, for the purpose of entrapping the nematodes and using them as a food source^39^. Additionally, the study revealed that the AWC neuron plays a large role in detection of MMB, and that multiple receptors may be activated by this odorant^39^. It is possible that prenol may be triggering similar neural circuitry, but further study is needed to verify if this is true, and explore if MMB has similar effects as prenol on the behavior of EPN IJs.

Despite all the previous mentions of both prenol and AMC, the exact source of these odorants in the context of EPN infections is unknown. It is conceivable that these odors could be produced by the EPNs themselves, the symbiotic bacteria that they carry, or as a byproduct of the decay of the insect cadaver. Further research is necessary to determine the origin of these compounds.

Our results showed that the response from IJs towards AMC was not as intense as the responses to prenol (Fig. 5C,D). Additionally, the lack of statistically significant differences in behavioral responses–by *S*. *glaseri* or *S*. *riobrave*–towards varying concentration of AMC, suggest that AMC may not be an important cue on its own (despite being associated with time points post-infection). However, it may be an important behavioral cue in combination with other cadaver-associated odors. It is possible that some cadaver-associated odors that interact with AMC or prenol are not highly volatile and therefore were not detected in the current study with SPME and GC-MS.

Conversely, prenol was highly repulsive to EPN IJs, while AMC was mildly repulsive at the highest concentration only. The behavioral responses of *S*. *glaseri* and *S*. *riobrave*, coupled to the association of prenol with a late time point in the infection suggests that prenol may be acting as an informative cue for free-living IJs that encounter an infected host. We speculate that prenol could act as a deterrent signal to free-living IJs and a dispersal cue for IJs within the decaying, resource-depleted insect cadaver, however further experimental evidence is needed to verify this hypothesis. Furthermore, we observed distinctly divergent behaviors between our EPNs and free-living nematodes such as *C*. *elegans* and *L*. *texanum* dauers, as well as the *D*. *melanogaster* larvae. We speculate that the behaviors observed are based on the niches that each organism occupies, but further study is needed. Additionally, the attraction of *D*. *melanogaster* larvae to prenol raises the question of whether prenol or other EPN-associated odors may be used not only to inform EPN IJs, but also potentially attract new hosts to depleted, EPN-filled cadavers. This remains to be explored. Additionally, the ability of prenol to overwhelm EPN IJ attraction to uninfected host odors, denotes a hierarchy of cues used to evaluate potential hosts. We speculate that prenol might be able to overcome other volatile cues that are generally associated with uninfected/healthy hosts, such as CO_2_.

## Conclusions

In this work, we have elaborated on the behaviors EPNs exhibit towards uninfected and infected hosts. This work has built upon prior evidence that EPNs exhibit different behavior towards naïve and infected hosts^13^, while evaluating possible mechanistic reasons as to how this occurs. Our work shows that behavioral responses (i.e., directional movement) of EPNs towards infected hosts are species-specific, and are, at least in part, mediated by volatile cues associated with the infected host. Among the volatiles identified from infected hosts, we found that prenol is strongly repulsive to the EPN species we tested, while it is attractive to organisms of different ecological niches (*C*. *elegans* and *L*. *texanum* dauers as well as *D*. *melanogaster* larvae). Prenol is an odorant we found associated with EPN infection and it reaches higher concentrations in late-stage infections. This indicates that prenol might be playing a multi-faceted role in both intra- and inter-specific scenarios, causing deterrence and dispersal for IJs, and attraction of potential hosts available in the vicinity. It may also be a food signal for bacterivorous nematodes such as *C*. *elegans*. Further investigation looking at a wider array of nematode species and potential hosts may shed light on the ecological relevance of prenol.

## Methods

### Nematode Culturing

*S*. *carpocapsae* was from inbred strain All, *S*. *glaseri* was from inbred strain NC, and *S*. *feltiae* was from inbred strain SN^40^. *S*. *riobrave* was from inbred strain TX-355^41^. EPN species were propagated as previously described^10, 11, 42^. Last-instar *Galleria mellonella* were infected with approximately 30 nematodes per host in a 6 cm petri dish and incubated at room temperature (Approximately 23 °C). After 7–10 days infected and deceased hosts were placed on white traps, which were incubated at room temperature. White traps were incubated for 7–10 days. IJs were collected from white traps, rinsed 3 times in tap water, and were placed in cell culture flasks. Collected IJs were kept at room temperature and used within two weeks of being collected (maximum age of 21 days post emergence). IJs were often used within a few days of being collected, with most assays using IJs that were 17 days post emergence or younger (used within 10 days of being collected). However, a few experiments were done using older IJs (14–21 days post emergence).

*Levipalatum texanum* ^20^ was isolated from *Scapteriscus borellii* mole crickets obtained through field sampling. Isolation of *L*. *texanum* was done using methods previously described and used to isolate *Pristionchus pacificus* from insects^43^. Briefly, mole crickets were sampled from Rio Hondo golf course in Downey, CA^11^. The mole crickets were cut in half longitudinally and laid on 2% agar plates. Nematodes observed on the plates after 1 week were isolated and cultured on nematode growth media plates (NGM) seeded with *E. coli* OP50, in the same manner as *C*. *elegans* (described below)^44^. Species identification was done by sequencing the 18S rRNA gene and identifying the closest match in GenBank. The 18S sequence we generated was nearly identical to the 18 S sequence from *L*. *texanum* EJR-2014, RS5280, accession number KJ877221 We used primers RH5401 and RH5402 to amplify and sequence the 5′ end and primers VL26345 and VL26346 to amplify and sequence the 3′ end of the small ribosomal subunit^45, 46^. Our sequences have been deposited in GenBank with accession numbers MF149117 and MF149118. *L*. *texanum* dauers were obtained using the same process used to obtain *C*. *elegans* dauers described below. We note that this is the first report of *L*. *texanum* coming from mole crickets, as they were originally isolated and described from scarab beetles.

*C*. *elegans* (strain N2) were cultured as previously described^44^ on NGM plates that had been seeded with OP50. To obtain dauers,  nematodes were transferred to NGM plates with thin lawns of OP50. Plates were left undisturbed for 10–14 days to allow the food supply to be depleted. These starved plates were then evaluated around 10–14 days after being seeded with adults. Dauer larvae were collected and rinsed 3 times in tap water before being stored in cell culture flasks at room temperature (approximately 23 °C) for storage and used within two weeks.

### Behavioral Response of IJs to Insect odors

Chemotaxis media plates were prepared as previously described^47^ and allowed to sit at room temperature for a minimum of 12 hours before use. Chemotaxis assays were performed as previously described^10, 11, 48^. 50 ml Hamilton gas-tight syringes were used. The test syringes were loaded with 5 *G*. *mellonella* larvae (uninfected or at various stages of infection), and control syringes left empty. The infected larvae used, were infected with conspecifics (e.g. *S*. *glaseri* IJs behavior was evaluated in response cadavers infected with *S*. *glaseri* IJs). The syringes were loaded into a KD Scientific pump (Model: KDS 220, Catalog number 78–0220NLSU).

Petri dish lids (from 100 mm plates) were modified as described. On either side of each lid two 10 mm holes were drilled approximately 10mm in from the edge. Nalgene PVC tubing (1/8″ diameter) was plugged into these holes to connect the plate lid to the syringes. This allowed air from the syringes to be deposited over the scoring circles of the scoring template, attached to the bottom of each chemotaxis plate. A pellet of approximately 250 IJs was deposited onto the center of the plate, and the plate oriented under the modified lid. The plate was oriented under the ports in the lid through which air from the syringes was being delivered. The plates were set on a vibration-reducing platform during the duration of the assay. Assays ran for approximately 1 hour (at a rate of 0.5 ml per minute). The assays were then scored using the scoring template placed on the bottom of each plate. A minimum of 3 experiments was done for each time point, and each experiment consisted of 9 technical replicates (minimum).

Chemotaxis index (CI) values were calculated^10, 11^. Briefly, we counted the number of nematodes inside each scoring circle on either side of the plate, over which odors from the test/host syringe or control syringe were being delivered. CI was calculated using the following equation: CI = # in host circle − # in control circle/Sum of all individuals in both circles.

Participation was calculated by counting the number of nematodes that moved directionally by 1 cm. Nematodes that did not move more than 1 cm out of the middle were scored as remaining in the middle section.

Statistical analyses were done using GraphPad PRISM software package. Chemotaxis index statistical analyses for responses to hosts over the course of the infection, was done using unpaired, ordinary, one-way ANOVA, along with Tukey’s (multiple comparisons) post-test (recommended by GraphPad PRISM). For age assays the collection of response to uninfected, 16 dpi and 200 mM prenol) was evaluated using unpaired two-way ANOVA with Sidak’s multiple comparisons posttest (as recommended by GraphPad PRISM). The age assay responses–of variable age, young and old–*S*. *glaseri* IJs to 16 dpi cadaver volatiles were additionally analyzed in isolation with one another with unpaired, ordinary one-way ANOVA with Tukey’s (multiple comparisons) post-test (as recommended by GraphPad PRISM).

Participation behavior was evaluated by statistically analyzing the data points within each group of “Host”, “Middle”, and “Control”. Analysis was done using unpaired, ordinary, one-way ANOVA (with recommended Tukey’s (multiple comparisons) post-test) to evaluate statistical differences between time points within each group. It should be noted that the statistical analysis for participation does not represent differences between the groups of Host, middle and Control.

### Gas-chromatography and mass-spectrometry analysis

GC-MS analysis procedure was based on Villaverde *et al*.^49^. Insects were incubated for 1 hour at room temperature in GC-MS vials (KaptClean Clear 27.5 mm by 95 mm, Part number GLA00797). The headspace volatiles were sampled for 30 minute at 25 °C using the solid phase microextraction fiber (65 µm PDMS/DVB fiber (Supelco Catalog number 57359-U)) exposed into the headspace of the sample vial through the septum cap. The chemicals sampled by the fiber were injected in Agilent Technologies 7890 A gas chromatograph equipped with a DB-5 column (30 m × 0.32 mm inner diameter, Agilent Technologies) in splitless mode, with the temperature program: 50 °C for 5 minutes, 5 °C/minute increase from 50 °C to 250 °C, and final hold at 250 °C for 10 minutes. The temperatures of the injector and transfer line were 250 °C. Helium was used as the carrier gas. Electron impact mass spectra (70 eV) were taken with an Agilent 5975C mass selective detector (Agilent Technologies).

Each different sample type was evaluated 3 separate times (on different days). Parameters for chemical candidates: appear 2 out of 3 runs, with an integration value of 10,000 or higher and a match score of 80% or higher (based on NIST 11 mass spectral library). Automatic peak integration of chromatograms was conducted using Enhanced Chem Station software: MSD Chemstation vE.02.02.1431 from Agilent Technologies. The RTE integrator was used, with the following parameters: Data point sampling was set to 5, Start threshold was set to 0.002 and stop threshold was set to 0. Output was set to calculate area counts.

For chemical identification, retention times and mass spectral data were compared between the synthetic standards and natural compounds detected from the infected-host samples. As the standards, prenol (under the name of 3-methyl-2-buten-1-ol) was purchased from Acros Organics and Acetyl methyl carbinol (AMC) (under the name Acetoin) was purchased from Tokyo Chemical Industry (TCI America). One microliter of the chemical was placed into a GC-MS vial (KaptClean Clear 27.5 mm by 95 mm, Part number GLA00797), and the vial was capped. After allowing the volatilization of the chemical in the vial for 1 hour, the headspace was sampled for 1–2 seconds with the solid phase microextraction fiber (65 µm PDMS/DVB fiber (Supelco Catalog number: 57359-U)).

### Behavioral Response of IJs to Chemical odorants

Chemical-response chemotaxis assays were also done as previously described^10, 11^. To either side of a chemotaxis plate (within the scoring circles) 2 µL of 1 M sodium azide was added, along with 5 µL of chemical odorant to the test circle side and 5 µL of diluent to the control circle. A pellet of approximately 250 nematodes was applied to the center of the plate and assays were allowed to run for 1 hour in the dark on a vibration-reducing platform. Hybrid assays combined use of syringe pump and chemical chemotaxis set-up.

Chemical odors and sodium azide were prepared as follows:

1 M AMC was prepared by dissolving 0.1762 grams in ultra-pure water (autoclaved, distilled, milli-Q filtered water). Ultra-pure water was used to make the serial dilution AMC. AMC was made and stored in glass vials (5/8^ths^ dram). Vials were wrapped in foil to prevent light exposure and were maintained in a plastic tip box kept at 4 °C while in storage to prevent degradation. Prepared chemical was used within 3 weeks.

2 M prenol was prepared by mixing 203 µL of 99.9% pure prenol with 797 µL of 100% ethanol. Ethanol was used to make a serial dilution series of prenol. Prenol was made and stored in glass vials (5/8^ths^ dram) in a plastic box to limit light exposure. Prenol was kept at room temperature and used within 3 weeks.

1 M sodium azide was prepared by dissolving 0.06501 grams of crystalline sodium azide in 1 mL of ultra-pure water. Sodium azide was prepared and stored in 1.5 mL plastic tubes at room temperature and used within 3 weeks.

### Behavioral response of *Drosophila melanogaster* to 200 mM prenol

Chemotaxis assays with *Drosophila melanogaster* larvae were modified from previously described assays^50–52^. Briefly, two PCR tube caps were placed on either side of a 1% agarose plate, in the center of the 2 cm scoring circles (template applied to the bottom of the plate was the same as those used in nematode behavior assays). We added 10 µL of 200 mM prenol diluted in paraffin oil to the PCR tube cap on the test side, while 10 µL of paraffin oil was added to the cap on the control side. The PCR cap ensures that the larvae are detecting prenol strictly as a volatile cue. Fruit flies (and fruit fly larvae) can sense chemical cues both through gustation and olfaction, unlike IJs, which have sealed buccal cavities, and perceive both volatiles and soluble chemical cues through their amphids^53^.

The plate was placed in a cardboard freezer box and left undisturbed for 5 minutes before being removed and a picture taken for scoring. The same template (as was used in nematode behavior assays) was used for scoring the larvae. Larvae were scored for a chemotaxis index (response index) as previously described in Monte *et al*.^50^. In addition we also collected data on participation to accurately represent the behavior of the *D*. *melanogaster* larvae.

### Hybrid Assays: Chemical-response Assays combined with Uninfected-host odors

Hybrid assays combined elements of the chemical response assay described above as well as the gas-tight syringes used in the host volatile assays. The syringes were used to deliver the volatiles from uninfected waxworms as described above. We applied prenol to the plate as previously described in the *Behavioral response of IJs to chemical odors* section above. 5 µL of prenol (Diluted in ultra-pure water to 2M) and ultra-pure water were added to the test and control sides of the plates respectively–within the scoring circles along with sodium azide as previously described. Approximately 250 IJs were applied to the center and the plate was placed under the modified lid as described in the *Behavioral response of IJs to Insect odors* section above. The assay ran for approximately 1 hour on an anti-vibration platform before being scored. Statistical analysis was done using GraphPad PRISM software package. We used a paired, two-tailed t-test for the analysis.

### Age Assays

Age assays testing insect odor were done as described above (in *Behavioral Response of IJs to Insect odors*) and assays using prenol were done as described above in *Behavioral Response of IJs to Chemical odorants*. IJs were cultured similarly as described above in *Nematode Culturing*, however culturing methods were changed slightly in the following ways: Infection incubation time was limited to 7 days, and white trap incubation was also limited to 7 days. IJs were washed as described before–in 3 washes of tap water, and stored in tap water at room temperature in cell culture flasks. *Young* IJs (1–8 days post emergence) were tested approximately 24 hours post collection, while *old* IJs (14–21 days post emergence) were tested 14 days post collection.

Statistical analyses were done using GraphPad PRISM software package. For CI and participation values in S1 and S3 unpaired two-way ANOVA was used with Sidak’s (multiple comparisons) post-test (recommended by PRISM). Statistical analysis of variable age IJs, young IJs and Old IJs (for *S*. *glaseri* IJ response to 16 dpi cadaver volatiles in S2) was done using unpaired, ordinary one-way ANOVA with Tukey’s (multiple comparisons) post-test (recommended by PRISM).

## Electronic supplementary material

Supplementary Information
