# Supplementary material for: Host seeking parasitic nematodes use specific odors to assess host resources
Source: Sci Rep. 2017 Jul 24;7:6270. doi: 10.1038/s41598-017-06620-2 (PMC5524962; doi:10.1038/s41598-017-06620-2)
Supplement: Supplementary file 1 — Supplementary Information [file 41598_2017_6620_MOESM1_ESM.doc]

**Host seeking parasitic nematodes use specific odors to assess host resources**

Tiffany Baiocchi^1^, Grant Lee^1^, Dong-Hwan Choe^2^, and Adler R. Dillman^1*^

^1^Department of Nematology, University of California, Riverside, California 92521, USA

^2^Department of Entomology, University of California, Riverside, California 92521, USA

^*^Corresponding author

**Supplemental Figures**

**
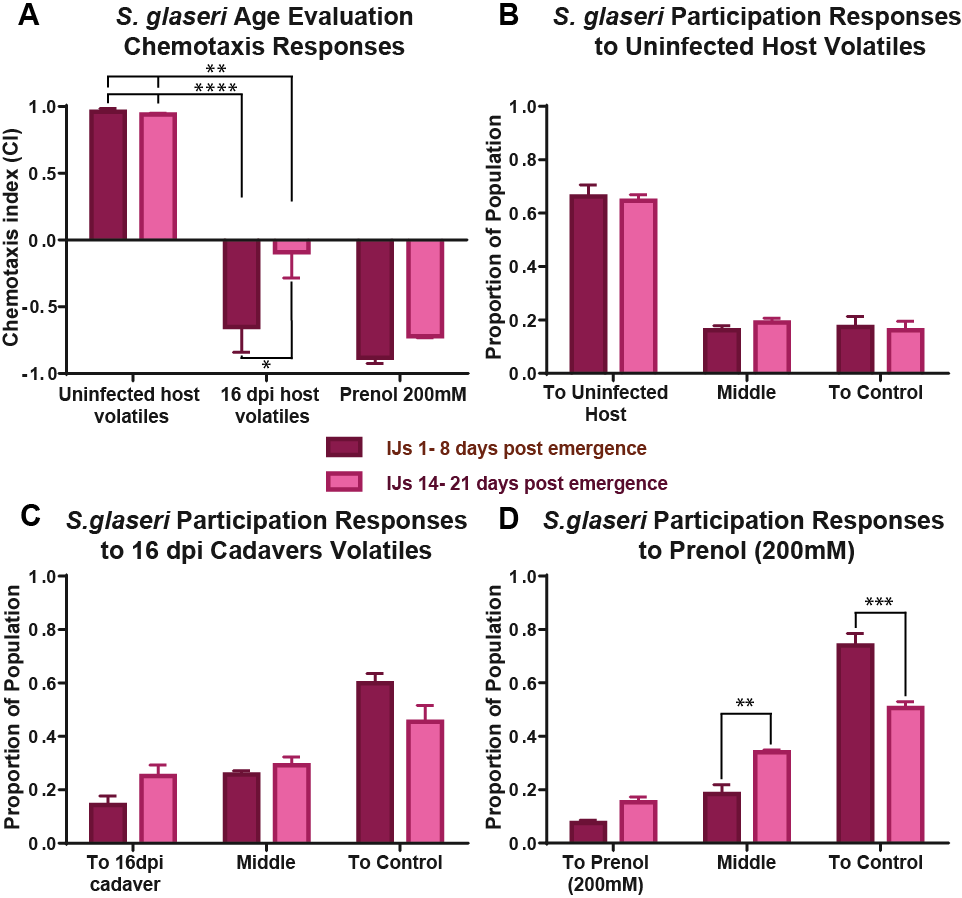
 Figure S1. Age Assay results for *S. glaseri*.** **(A)** Chemotaxis indices for the age assays for responses to uninfected, 16 dpi cadavers infected with *S. glaseri* and 200mM prenol. There was statistical significance between the two age groups for 16 dpi cadaver volatiles only. For responses to uninfected host volatiles and response to 200mM prenol there was no significant differences in behavioral. **(B)** Participation results for responses to uninfected host volatiles showed no significant differences in behavior between IJ age groups. **(C)** Participation results for IJs responding to 16dpi cadaver volatiles showed no significant differences in behavior between IJ age groups. **(D)** Participation results for IJs responding to prenol. Results indicated a significant increase in portion of older IJs that remained in the middle, as well as a significantly reduction in proportion of IJs that traveled towards the control. Statistical analysis- for both CI and participation results- was done using unpaired two-way ANOVA. Error bars represent SEM.


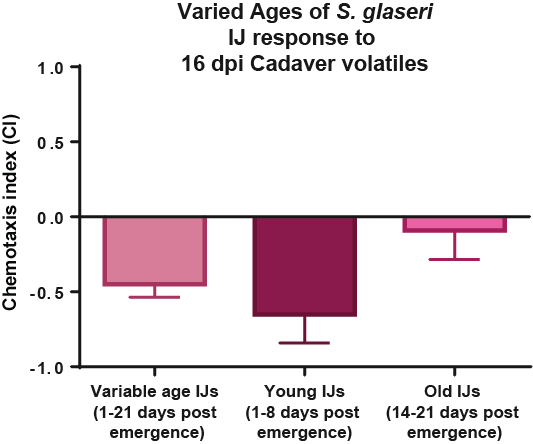


**Figure S2. Age Assay results for *S. glaseri* response to 16 dpi by variable age, young and old IJs.** For experiments done to test *S. glaseri* responses to host volatiles throughout the course of infection the age range of IJs was between 1-21 days post-emergence. Chemotaxis indices above are a comparison of IJ responses to 16 dpi cadavers from variable ages (1-21 days post emergence), young IJs (1-8 days post emergence) and old IJs (14-21 days post emergence). Statistical analysis was done using unpaired, ordinary, one-way ANOVA (with Tukey’s multiple comparisons post-test). No significant difference was found between the Variable age CI and the CI values from either young or old IJs. Additionally the statistical analysis revealed there is no significant difference between young and old IJ responses to 16 dpi cadaver volatiles. Error bars represent SEM.


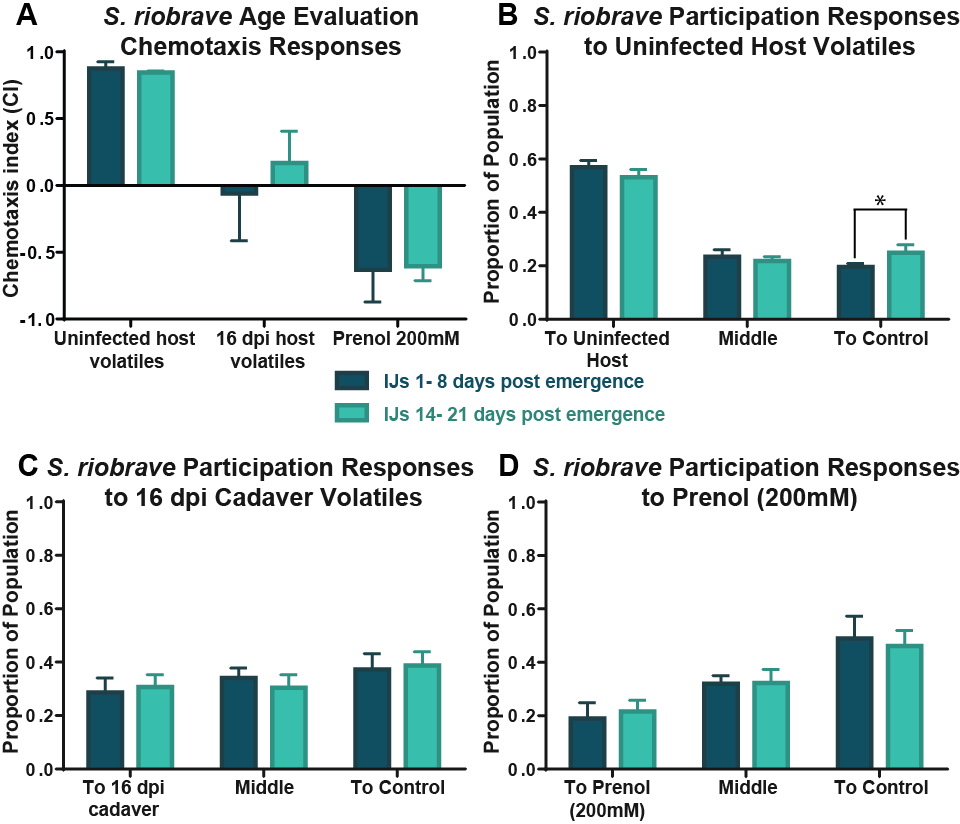


**Figure S3. Age Assay results for *S. riobrave*.** **(A)** Chemotaxis indices for the age assays for responses to uninfected, 16 dpi cadavers infected with *S. riobrave* and 200mM prenol. There was no statistical significance between the two age groups for any of the categories tested. **(B)** Participation results for responses to uninfected hosts. For the proportion of the populations that traveled towards the host or remained in the middle there was no statistical significance. The portion heading away from the uninfected host (to control) we observed an increase for the older IJs. **(C)** Participation results for IJs responding to 16dpi cadaver volatiles showed no significant differences in behavior between IJ age groups. **(D)** Participation results for IJs responding to prenol we saw no significant differences in behavior between age groups. Statistical analysis- for both CI and participation results- was done using unpaired two-way ANOVA. Error bars represent SEM.


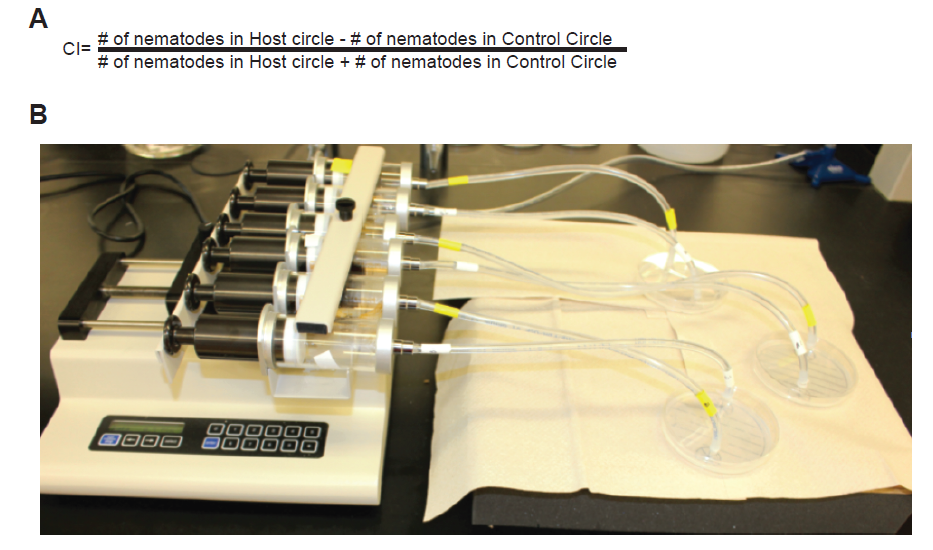


**Figure S4. A)** The equation used to calculate chemotaxis index. **B)** A photograph of the syringe-pump system set up with syringes loaded with uninfected *Galleria mellonella*. PVC tubing is attached to the end of the syringe and delivers air from the syringe to the chemotaxis plates. Yellow tape marks tubes connected to syringes with hosts, while white tape denotes tubes attached to blank (control) syringes.
